# Case Report: Cumulative proton dose reconstruction using CBCT-based synthetic CT for interfraction metallic port variability in breast tissue expanders

**DOI:** 10.3389/fonc.2023.1132178

**Published:** 2023-07-27

**Authors:** Chin-Cheng Chen, Jiayi Liu, Peter Park, Andy Shim, Sheng Huang, Sarah Wong, Pingfang Tsai, Haibo Lin, J. Isabelle Choi

**Affiliations:** ^1^ New York Proton Center, New York, NY, United States; ^2^ Institute of Nuclear Engineering and Science, National Tsing Hua University, Hsinchu, Taiwan; ^3^ Memorial Sloan-Kettering Cancer Center, New York, NY, United States; ^4^ Department of Radiation Oncology, Montefiore Medical Center and Albert Einstein College of Medicine, Bronx, NY, United States

**Keywords:** proton, tissue expander, CBCT-based synthetic CT, breast cancer, dose reconstruction and dosimetric impact

## Abstract

**Introduction:**

Dose perturbation of spot-scanning proton beams passing through a dislocated metallic port (MP) of a breast tissue expander may degrade target dose coverage or deliver excess dose to the ipsilateral lung and heart. The feasibility of utilizing daily cone-beam computed tomography (CBCT)–based synthetic CTs (synCTs) for dose reconstruction was evaluated, and the fractional and cumulative dosimetric impact due to daily MP dislocation is reported.

**Methods:**

The synCT was generated by deforming the simulation CT to daily CBCT. The MP structure template was mapped onto all CTs on the basis of daily MP position. Proton treatment plans were generated with two and three fields on the planned CT (pCT, Plan A) and the first verification CT (vCT, Plan B), respectively, for a fractional dose of 1.8 Gy(RBE). Plan A and Plan B were used alternatively, as determined by the daily MP position. The reconstructed fractional doses were calculated with corresponding plans and synCTs, and the cumulative doses were summed with the rigid or deformed fractional doses on pCT and vCT.

**Results:**

The planned and reconstructed fractional dose demonstrated a low-dose socket around the planned MP position due to the use of field-specific targets (FSTs). Dose hot spots with >120% of the prescription due to MP dislocation were found behind the planned MP position on most reconstructed fractional doses. The reconstructed cumulative dose shows two low-dose sockets around the two planned MP positions reflecting the two plans used. The doses at the hot spots behind the planned MPs averaged out to 114% of the prescription. The cumulative D_95%_ of the CTV_Chest Wall decreased by up to 2.4% and 4.0%, and the cumulative V_20Gy(RBE)_ of the left lung decreased to 16.1% and 16.8% on pCT and vCT, respectively. The cumulative D_mean_ of the heart decreased to as low as 0.7 Gy(RBE) on pCT but increased to as high as 1.6 Gy(RBE) on vCT.

**Conclusion:**

The robustness of proton plans using FSTs around the magnet in the MP of the tissue expander can be improved by applying multiple fields and plans, which provides forgiveness of dose heterogeneity incurred from dislocation of high-Z materials in this single case.

## Introduction

Proton therapy used for breast cancer treatments is becoming more prevalent as access to proton centers increases globally (1). The *en face* beams used in most proton treatment plans for breast cancers provide a homogeneous and conformal dose to the clinical target volume (CTV) while sparing the heart and lung beyond the sharp dose falloff at Bragg peaks. Patients with breast cancer with tissue expanders who have undergone mastectomy with plan for two-stage reconstruction could be also treated with proton beams (2–6). In this approach, a saline-filled tissue expander with an embedded metallic port (MP) for fluid injection is placed at the time of mastectomy. The MP is usually constructed of a magnet enclosed in a metal case that acts as a needle guard (3).

Different planning techniques for patients with breast cancer with tissue expanders using spot-scanning proton beams have been reported (2–6). Spot-scanning proton beams can be used to shoot through the MP in the tissue expander with accurate Monte Carlo dose calculation (2) or pencil-beam convolution algorithm with well modeled and validated geometries and materials of MP (3). Spot-scanning proton beams can also be used to shoot around the MP in tissue expanders (4–7). Kirk et al. (4) and Zhu et al. (5) reported on the application of field-specific targets (FSTs) to avoid spot placements inside and beyond the MP. Two to three proton fields are used in either technique to achieve a proton treatment plan with maximal robustness.

The MP in the tissue expander requires careful delineation on computed tomography (CT) images, and the stopping power of the MP materials should be assigned accurately. Although metal artifact reduction algorithms can be used to reduce artifacts significantly, streak artifact caused by the magnet remains visible. Fortunately, both the physical geometries and materials of the MP can be provided by major manufacturers. A template of the MP can be constructed on the basis of manufacturer’s specifications and mapped on patients’ CT images. MP displacement during treatment should also be considered. Mutter et al. reported that MP location is within a 1-cm difference from the planned CT (pCT) position for >95% of treatment fractions and that the dosimetric impact was clinically acceptable considering both CTV coverage and normal tissue (heart and ipsilateral lung) sparing with a 1-cm offset in the worst-case scenarios (2). However, the dosimetric impact of MP dislocations larger than 1 cm from its planned position is rarely reported in publications. Dose delivery of proton beams passing through a dislocated MP may either degrade target dose coverage or overdose the ipsilateral lung and heart.

A left-sided postmastectomy patient with Allergan Natrelle^®^ 133 tissue expander (Allergan, Inc., Dublin, Ireland) was planned with a two-field beam arrangement with FSTs (Plan A) around the MP for a prescription of 50.4 Gy(RBE) in 28 fractions. The MP was found dislocated on the first day of treatment, and a verification CT (vCT) scan was performed for plan revision (Plan B). However, the MP on subsequent fractions was found to be relocated daily with more than 5-mm displacements compared with the planned positions of either Plan A or Plan B. The patient was then treated with Plan A or Plan B alternatively, as determined by the daily MP position shown on X-ray images, which left the daily and cumulative doses unknown due to the daily variations in the MP position.

Veiga et al. first proposed the “dose of the day” reconstruction using CT–to–cone-beam CT (CBCT) for head and neck patients treated with photon intensity modulated radiation therapy by deforming a pCT to match a daily CBCT (7). They later demonstrated the proton dose calculation on virtual CT by deforming the pCT onto the daily CBCT for adaptive proton therapy of lung cancer, in which the virtual CT was also corrected for anatomy change such as pleural effusion and tumor regression (8). The daily CBCTs of the breast patient could represent the real-time position and the anatomy change including the MP dislocation during daily treatments. The deformed reference CT onto the daily CBCT, or CBCT-based synthetic CT (synCT), with manual correction of the MP position, can be used for daily and cumulative dose reconstruction.

In this study, the feasibility of utilizing daily CBCT-based synCTs for proton dose reconstructions was evaluated. The CBCT-based synCTs for 28 fractions were generated with the dislocated MP. The reconstructed fractional doses were calculated with corresponding plans and synCTs, and the cumulative doses were summed with rigid or deformed fraction doses to evaluate the dosimetric impact due to daily MP dislocations.

## Methods

### Patient selection

A 33-year-old female patient diagnosed with left breast cancer, clinical stage T2N0, underwent bilateral mastectomy with immediate tissue expander (Natrelle^®^ Allergan 133) reconstruction, with surgical pathology demonstrating pathologic stage T2N1 disease (2.4-cm primary tumor, 2/4 involved sentinel lymph nodes), grade 3; with lymphovascular invasion, estrogen receptor positive, progesterone receptor positive, and human epidermal growth factor receptor 2 (HER-2) negative; and with negative surgical margins. She received spot-scanning proton therapy for her adjuvant radiation therapy with a prescription of 50.4 Gy(RBE) in 28 fractions to the left reconstructed chest wall and comprehensive regional lymph nodes. This retrospective study is approved (NYPC ERC# 2020-026) by the Western Institutional Review Board, Inc. (Puyallup, WA, USA).

### Simulated Planned CT and verification CT

The patient was positioned head-first supine, with head turned to the right and both arms placed above the head, immobilized with VacQfix™ Vacuum Cushions (Qfix, Avondale, PA, USA). The pCT was acquired 2 weeks prior to the first patient treatment, and the vCT was acquired on the first day of patient treatment with the same patient set up when the MP was found dislocated. Both pCT and vCT were acquired by SIEMENS SOMATOM Definition Edge CT scanner (Siemens Healthcare GmbH, Germany) with slice thickness of 2 mm in a scanning range from bottom of orbits to L2 spine.

### CBCT-based synthetic CT

The CBCT-based synCT was generated in the Velocity™ Oncology Imaging Informatics System (Varian Medical Systems, Palo Alto, CA, USA) by deforming either the pCT or vCT to daily CBCT. Limited by the field-of-view (FOV) of the image panel used, only 20 cm length in patient’s superior-to-inferior direction around the treatment isocenter of the pCT and vCT can be deformed to CBCT. The CTs combining the CBCT-based synCTs in the FOV and the reference CTs outside the FOVs were then used for dose reconstruction.

### Metallic port template inserted on CTs

**Figure 1:**
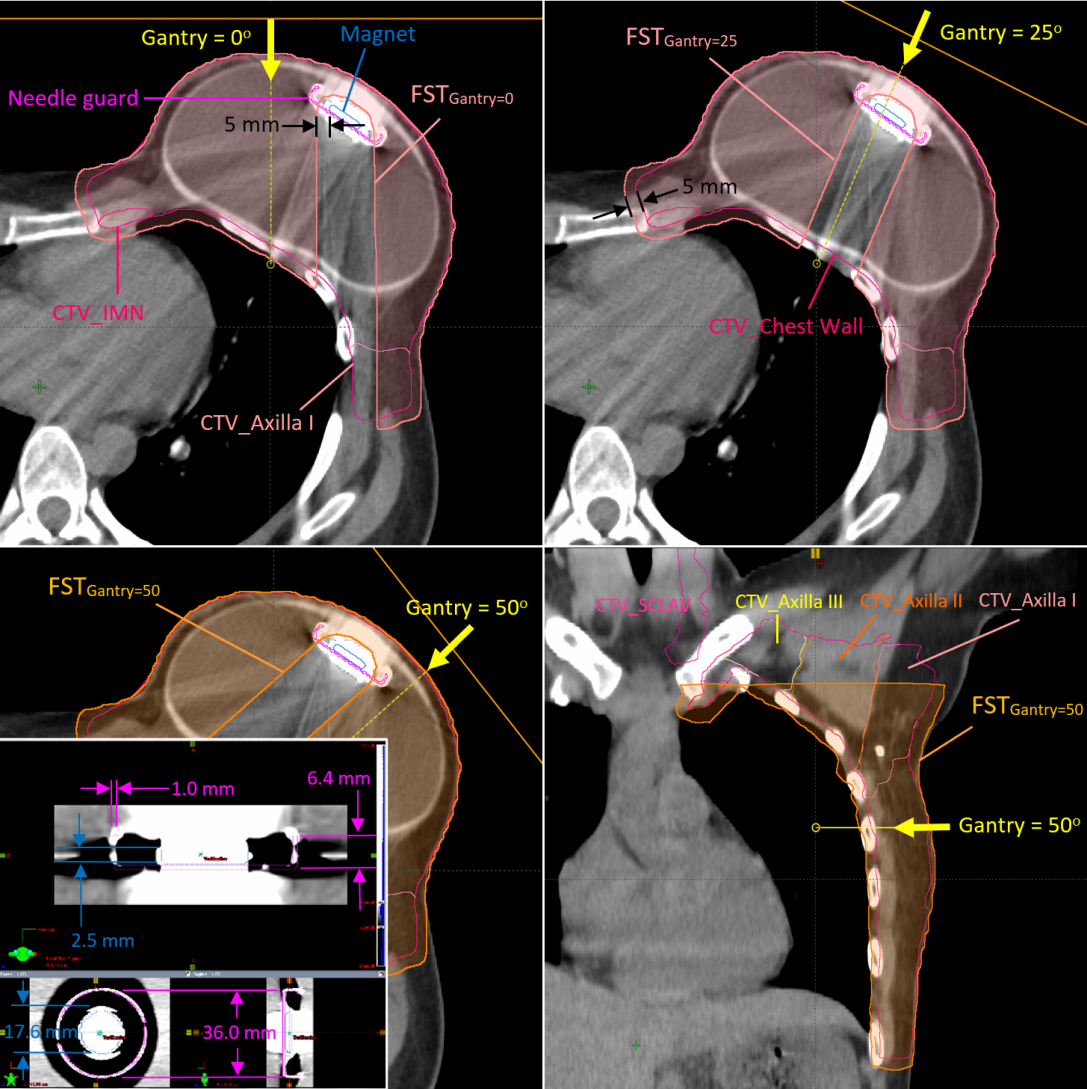
Clinical target volumes (CTVs) and the field-specific targets used for the fields at gantry angles of 0°, 25°, and 45° in Plan B The metallic port template in high-resolution CT image is also shown at the left-bottom corner of the figure.

An MP structure template including the magnet and metal case as the needle guard on high-resolution CT images was delineated on the basis of the manufacturer’s specifications and is used in our clinics routinely for patients with breast cancer with Natrelle^®^ Allergan 133 tissue expander. The MP structure template on a high-resolution CT image is shown in **Figure 1**. The magnet in the Natrelle^®^ 133 is Samarium Cobalt alloy with a mass density of 8.4 g/cm^3^ per manufacturer, which is identical with the mass density of brass. The relative linear stopping power (RLSP) of the brass is 5.71, which is also close to the RLSP of 5.5 measured by the Mayo group (2). A Hounsfield unit (HU) of 9,316 was assigned to the magnet using our institutional calibration curve, converting the HU to RSLP. The needle guard encapsulating the magnet is made by titanium alloy with an RLSP of 3.17, and a HU of 4,540 was assigned. The water-equivalent thickness (WET) of the magnet and base of the needle guard are 13.8 mm and 3.17 mm respectively. The outline of saline-filled tissue expander was also contoured, and the HU of saline inside the tissue expander was overridden with the RLSP of 1.0. The MP structure was copied onto all CTs (pCT, vCT, and CBCT-based synCT) after the templated CT was rigid registered with the target CT images.

### Treatment plans

The CTV was delineated using the RadComp contouring atlas and included the left chest wall and regional nodes (axilla, internal mammary, and supraclavicular nodes) (9). The proton spot-scanning treatment plans were generated with two (G0° and G45°) and three (G0°, G25°, and G50°) fields on the pCT (Plan A) and the first vCT (Plan B), respectively, for the fractional dose of 1.8 Gy(RBE) using Eclipse treatment planning system (TPS) (Varian Medical System, Palo Alto, CA, USA, version 15.6). Additional fields and different gantry angles were used in Plan B to improve the overall robustness of Plan A (a total four different gantry angles using five different FSTs). **Figure 1** shows the FSTs with 5-mm geometrical margin from the magnet to avoid heavily weighted protons passing through the high-Z material in Plan B. The FSTs were also expanded with a 5-mm geometrical margin outwardly and cropped the patient body. It allows some spots placed at the peripheral dose falloff around the CTV and provides more flexibility for the optimizer to avoid hot spots at the edge of CTV. Another 1 mm in WET was applied in the axial margin at the distal end of all FSTs, which serves the same purpose to avoid the dose spike at the distal end of the spread-out Bragg peaks, especially at the rib cage. The FSTs for larger gantry angles such as 45° (Plan A) and 50° (Plan B) were cropped superiorly to avoid the proton beam shooting through the left arm. Both Plan A and Plan B were generated with multi-field optimization and robust optimization with ±5-mm setup and ±3.5% range uncertainties. As determined by the daily position of the MP, the patient received 17 fractions from Plan A and 11 fractions from Plan B.

### Patient treatments and dose reconstructions

The patient was treated with either Plan A or Plan B, as determined by the MP positions on 2D kilovolt (kV) images taken prior to the CBCT. The fractional doses were forward calculated with Plan A (17 fractional doses) or Plan B (11 fractional doses) on the CBCT-based synCT generated using pCT or vCT correspondingly. The cumulative doses were generated with rigid or deformable dose sum. The rigid dose sum was the direct sum of the reconstructed fractional doses using daily CBCT registration vector in Eclipse TPS. The rigid dose sum was then projected on either pCT or vCT for further evaluation. All reconstructed fractional doses were also deformed onto pCT and vCT using MIM Software (version 7.2.7, MIM Software, Inc., OH), respectively. The deformed fractional doses were then summed as the cumulative doses on pCT and vCT.

## Results

### Fractional doses

**Figure 2:**
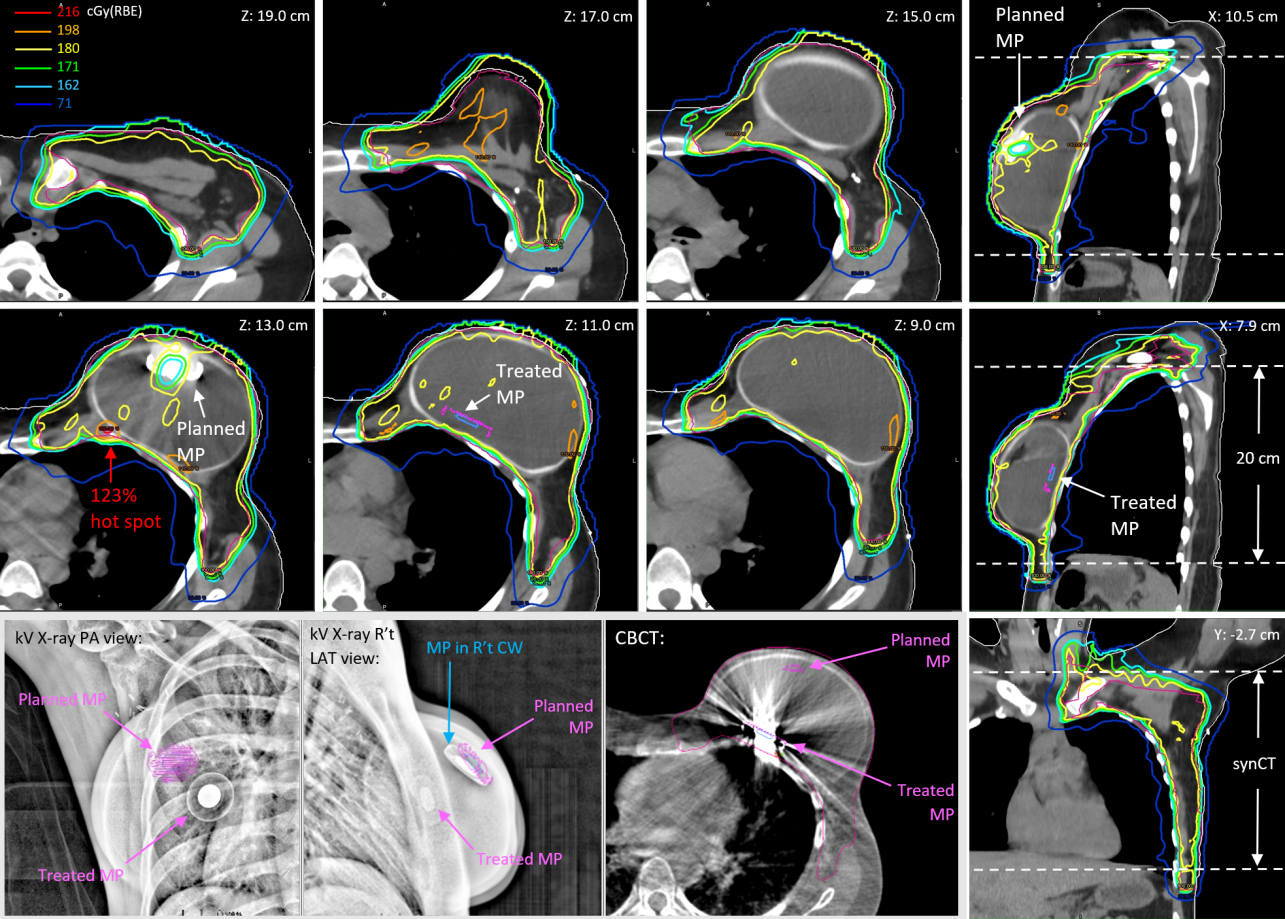
The reconstructed fractional doses on the day of 19 July 2021 for a prescription of 180 cGy(RBE) per fraction. The kV X-ray images and CBCT show the dislocation of the treated MP versus the planned MP.

**Figure 2** shows the planned MP contours projected on the kV X-ray and CBCT images with the displaced MP on 19 July 2021 when the patient was treated with Plan A and the reconstructed fractional dose on the synCT. The MP artifact shown at Z = 13.0 cm could not be removed when the pCT was deformed to CBCT. Consequently, the artifact around and including the planned MP with high HU was then assigned as saline (RLSP = 1.0) in the forward calculation. The treated MP inserted as described in Methods and shown at Z = 11.0 cm and X = 7.9 cm in **Figure 2** was calculated. The low-dose socket around the planned MP due to the use of FSTs is distorted slightly, and the protons at the edge of FSTs around the planned MP were over-ranged due to the absence of the MP from its planned position. A significant dose hot spot of 220.9 cGy(RBE) (123% of the prescribed fractional dose) was found close to the rib cage (Z = 13.0 cm). The displaced MP at Z = 11.0 cm moved into FSTs and pulled back the proton ranges, which caused some small cold spots [yellow circle for doses <180 cGy(RBE)] inside the CTV. In addition to the MP displacement, the shape of the tissue expander changed slightly, and some setup discrepancies were found at the discontinuity of the limited synCT FOV edge. Consequently, small cold spots (<100% of the prescribed fractional dose) were found in the superior and inferior part of the CTV.

### Cumulative doses

**Figure 3:**
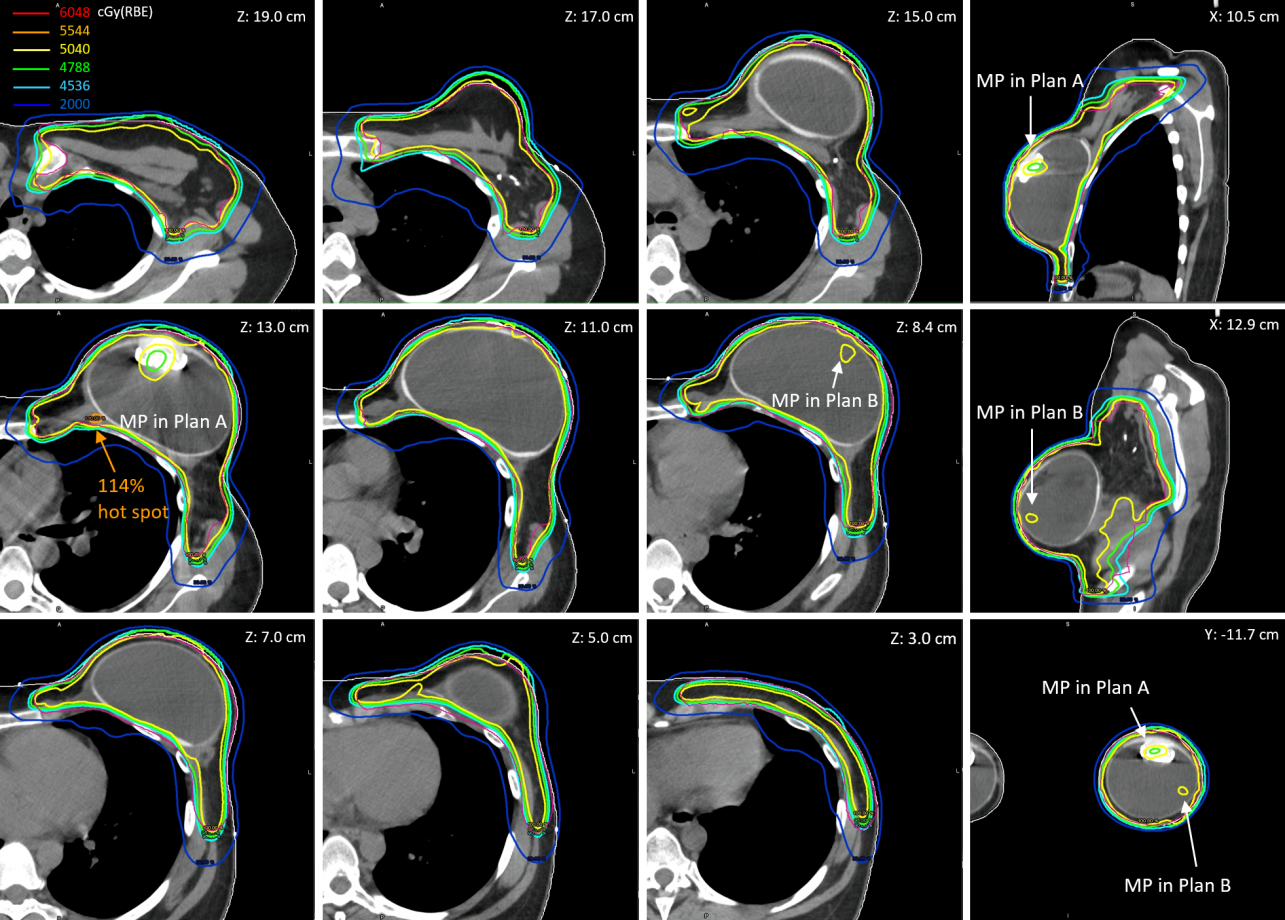
The reconstructed cumulative doses as the sum of deformed fractional doses (17 fractions from Plan A and 11 fractions from Plan B) on pCT.

**Figure 3** shows the reconstructed cumulative doses as the sum of deformed fractional doses on pCT. The low-dose socket on fractional dose maintained when the MP moved away from the planned positions. Consequently, there were two low-dose sockets around the two planned MP positions on the reconstructed cumulative doses (Z = 13.0 cm and Z = 8.4 cm). Hot spots due to the absence of planned MP on fractional doses were also averaged out because of the two planned MP positions in the cumulative doses. The 114% global hot spot was found at Z = 13.0 cm behind the planned MP position in Plan A, in which 17 of the 28 fractions were used. 

The dose–volume histogram metrics of planned and reconstructed cumulative doses on pCT and vCT are listed in **Table 1**. The cumulative D_95%_ of the CTV_Chest Wall decreased by up to 2.4% (rigid) and 4.0% (deformed) on pCT and vCT, respectively, from 98.8% in the nominal plans, due to the two low-dose sockets in two plans. The low-dose sockets were around the planned magnets and inside the tissue expander where no tumor cells were involved. As mentioned in the reconstructed fractional doses, the MP moved into the FSTs and then pulled back the proton ranges, which also pulled back the 20 Gy(RBE) isodose lines in the left lung. The relative volume of the left lung receiving at least 20 Gy(RBE) decreased by 3%–4% in reconstructed cumulative doses. The V_20Gy(RBE)_ of left lung were to 16.1% (deformed) and 16.8% (rigid) on pCT and vCT, respectively, compared with 19.8% and 19.4% in the planned doses. The cumulative D_mean_ of the heart decreased to as low as 0.7 Gy(RBE) on pCT but increased to as high as 1.6 Gy(RBE) on vCT when the rigid plan sums were considered.

**Table 1:** Comparisons of dose metrics of the planned and reconstructed (rigid or deformed) cumulative doses.

Structure	Dose Metric	Dose on pCT			Dose on vCT		
		Planned	Rigid	Deformed	Planned	Rigid	Deformed
CTV_50.4	D_95%_ (%)	99.2	96.3	96.7	99.1	95.2	96.4
	D_max_ (%)	113.7	115.1	113.7	113.9	115.3	113.2
CTV_Chest Wall	D_95%_ (%)	98.7	96.8	96.3	98.8	94.8	96.0
	D_max_ (%)	113.2	115.1	113.7	113.9	115.3	113.2
CTV_Axilla I L	D_95%_ (%)	100.9	97.0	98.8	100.7	97.7	98.3
CTV_Axilla II L	D_95%_ (%)	101.1	96.9	96.4	101.0	98.7	97.1
CTV_Axilla II L	D_95%_ (%)	101.4	98.5	99.8	101.2	101.1	100.8
CTV_SCLAV L	D_95%_ (%)	100.2	92.4	99.7	99.3	92.4	99.7
CTV_IMN	D_95%_ [Gy(RBE)]	50.2	44.9	46.9	49.8	49.9	48.2
Left Lung	V_20Gy(RBE)_ (%)	19.8	15.3	16.1	19.4	16.8	16.0
Heart	D_mean_ [Gy(RBE)]	1.0	0.7	1.0	1.0	1.6	0.9
Esophagus	D_max_[(Gy(RBE)]	33.8	31.2	32.9	38.2	37.9	35.9
Spinal Cord	D_max_ [Gy(RBE)]	7.1	5.6	7.7	5.9	6.2	5.6

## Discussion

Dose reconstruction using daily CBCT-based synCTs was demonstrated in this study. The synCT is the deformation of the referenced CT on to the daily CBCT with on-line image registration, which represents the most realistic patient setup during beam delivery.

The high-Z materials and resultant artifacts with high HU values on the referenced CT (pCT and vCT) cannot be deformed correctly onto daily CBCT. Consequently, the planned MP and artifact on synCT require removal by assigning appropriate RLSPs, and the MP structure template had to be manually inserted onto the synCT based on the daily MP location on CBCT as shown on **Figure 2** (Z = 11.0 cm). The MP template insertion was the most time-consuming step. All the structure delineations and HU overrides were checked carefully before forward calculating the fractional doses.

Because of the physical limitation of the image panels on the treatment nozzle, only 20-cm FOV can be acquired in a single scan of CBCT. The treatment isocenter is selected as the geometrical isocenter of the whole CTV (chest wall and all regional nodes) in our current practice, and the FOV captures majority of CTV_Chest Wall, where the tissue expander is located. A small part of the regional node CTVs and inferior lungs were missed on the CBCT as shown in **Figure 2** (coronal view). The reconstructed fractional doses in the missed regions would be identical with the planned doses and thus underestimate the dosimetric impact of patient setup in dose delivery.

The reconstructed fractional dose shows the most realistic daily dose delivery but in a single fraction. The D_95%_ of the CTV_Chest Wall ranged from 90.5% to 97.6% with an average of 95.5% for 28 fractions. The highest heart mean dose [equivalent to 1.43 Gy(RBE) with 28 fractions] was found on the very first day of patient treatment when the MP was found displaced and the revised plan (Plan B) was not ready. The V_71cGy(RBE)_ of left lung [equivalent to V_20Gy(RBE)_ with 28 fractions] could be as low as 12.9% or as high as 20.8% with an average of 16.4%. The fractional dose sums were projected on either pCT or vCT to evaluate the cumulative dose impact due to the MP variability in position. However, the analysis of dose–volume histogram metrics relied on the patient anatomy on a single CT. The lowest D_95%_ of the CTV_Chest Wall was 94.8% with rigid dose sum on vCT, which was comparable with the average D_95%_ from the fractional dose distribution. The results of the delivered V_20Gy(RBE)_ of the left lung are lower than the planned value because the displaced MP pulled back the proton range in the beam path unexpectedly. The heart D_mean_ of 1.6 Gy(RBE) was found in the cumulative dose with rigid plan sum on vCT. The vCT shows a lower tissue expander and an increased contact between the heart and chest wall. However, the deformed plan sum on pCT and vCT show heart D_mean_ equal or less than 1.0 Gy(RBE). A rigid plan sum projected on an unfavorable anatomy could overestimate the dosimetric impact.

A total of four gantry angles with five FSTs in two plans provided robust dose coverage of the CTV regardless of MP displacement. Dose coverage did not degrade significantly behind the unexpected MP position. The global D_max_ up to >120% found on the most reconstructed fractional doses behind the planned MP positions was averaged out in cumulative doses with two plans.

## Conclusion

CBCT-based synCT can be used to reduce the frequency of verification CTs, especially for patients with breast cancer who will likely not experience significant toxicity-related weight loss or change in tumor size compared with other treatment sites. Dose reconstruction using synCTs associated with online image registration represents daily dose delivery on the most realistic patient setup. However, because of the physical limitation of the FOV of the CBCT, only the doses of targets and normal tissues inside the FOV can be evaluated. Robustness of proton plans using FSTs around the magnet in the MP of tissue expanders can be improved with multiple fields and plans, which provides forgiveness of dose heterogeneity incurred from dislocation of high-Z materials.

## Author’s note

Presented in part at American Association of Physicists in Medicine, Spring Clinical Meeting, March 26-29, 2022, New Orleans, LA.

## Data availability statement

The original contributions presented in the study are included in the article/supplementary material. Further inquiries can be directed to the corresponding author.

## Ethics statement

The studies involving human participants were reviewed and approved by Western Institutional Review Board, Inc. (NYPC ERC# 2020-026). Written informed consent was obtained from the participant/patient(s) for the publication of this case report.

## Author contributions

Conceptualization, C-CC and JL; methodology, C-CC and JL; software, C-CC, PP, JL, SH and PT; patient treatment, SW; resources, AS and HL; writing—original draft preparation, C-CC; writing—review and editing, JC. C-CC and JL contributed equally to this manuscript. All authors have read and agreed to the published version of the manuscript.
